# Pericarditis and Autoinflammation: A Clinical and Genetic Analysis of Patients With Idiopathic Recurrent Pericarditis and Monogenic Autoinflammatory Diseases at a National Referral Center

**DOI:** 10.1161/JAHA.121.024931

**Published:** 2022-06-06

**Authors:** Claire J. Peet, Dorota Rowczenio, Ebun Omoyinmi, Charalampia Papadopoulou, Bella Ruth R. Mapalo, Michael R. Wood, Francesca Capon, Helen J. Lachmann

**Affiliations:** ^1^ National Amyloidosis Centre Royal Free London NHS Foundation Trust & Division of Medicine University, College London London United Kingdom; ^2^ Department of Medical and Molecular Genetics King’s College London London United Kingdom

**Keywords:** autoinflammation, inflammation, pericarditis, Pericardial Disease, Inflammation, Genetic, Association Studies

## Abstract

**Background:**

Idiopathic recurrent pericarditis (IRP) is an orphan disease that carries significant morbidity, partly driven by corticosteroid dependence. Innate immune modulators, colchicine and anti‐interleukin‐1 agents, pioneered in monogenic autoinflammatory diseases, have demonstrated remarkable efficacy in trials, suggesting that autoinflammation may contribute to IRP. This study characterizes the phenotype of patients with IRP and monogenic autoinflammatory diseases, and establishes whether autoinflammatory disease genes are associated with IRP.

**Methods and Results:**

We retrospectively analyzed the medical records of patients with IRP (n=136) and monogenic autoinflammatory diseases (n=1910) attending a national center (London, UK) between 2000 and 2021. We examined 4 genes (*MEFV, MVK, NLRP3, TNFRSF1A*) by next‐generation sequencing in 128 patients with IRP and compared the frequency of rare deleterious variants to controls obtained from the Genome Aggregation Database. In this cohort of patients with IRP, corticosteroid dependence was common (39/136, 28.7%) and was associated with chronic pain (adjusted odds ratio 2.8 [95% CI, 1.3–6.5], *P*=0.012). IRP frequently manifested with systemic inflammation (raised C‐reactive protein [121/136, 89.0%] and extrapericardial effusions [68/136, 50.0%]). Pericarditis was observed in all examined monogenic autoinflammatory diseases (0.4%–3.7% of cases). Rare deleterious *MEFV* variants were more frequent in IRP than in ancestry‐matched controls (allele frequency 9/200 versus 2932/129 200, *P*=0.040).

**Conclusions:**

Pericarditis is a feature of interleukin‐1 driven monogenic autoinflammatory diseases and IRP is associated with variants in *MEFV*, a gene involved in interleukin‐1β processing. We also found that corticosteroid dependence in IRP is associated with chronic noninflammatory pain. Together these data implicate autoinflammation in IRP and support reducing reliance on corticosteroids in its management.

**Nonstandard Abbreviations and Acronyms**
CAPS: cryopyrin associated periodic syndrome
ESC: European Society of Cardiology
FMF: familial Mediterranean fever
gnomAD: genome aggregation database
IRP: idiopathic recurrent pericarditis
***MEFV***
: MEFV innate immunity regulator, pyrin
MKD: mevalonate kinase deficiency
***MVK***
: mevalonate kinase
***NLRP3***
: NOD‐like receptor family pyrin domain containing 3
SAID: systemic autoinflammatory disease
***TNFRSF1A***
: TNF receptor superfamily 1A
TRAPS: TNF receptor associated periodic syndrome

**Clinical Perspective**

**What Is New?**

- This study characterizes the clinical features of patients with idiopathic recurrent pericarditis (IRP), evaluates the prevalence of pericarditis in monogenic autoinflammatory diseases, and investigates whether variants in genes implicated in autoinflammation are associated with IRP.
- Corticosteroid dependence in IRP was associated with noninflammatory chest pain and chronic fatigue.
- IRP frequently presented with extrapericardial inflammation, and IRP was associated with the presence of rare deleterious variants in the *MEFV* gene, which is involved in interleukin‐1β processing; pericarditis was also seen in all 4 monogenic interleukin‐1‐mediated autoinflammatory diseases.

**What Are the Clinical Implications?**

- Corticosteroid dependence is associated with poor outcomes in IRP, illustrating the need for alternate, targeted treatment approaches.
- IRP shares clinical and genetic features with the IL‐1‐mediated systemic autoinflammatory diseases, where use of targeted immunomodulation, with colchicine and anti‐IL‐1 agents, has revolutionized prognosis.
- Accordingly, these data support increased uptake of these agents and a reduced reliance on corticosteroids in the management of IRP.

Idiopathic recurrent pericarditis (IRP) is a poorly understood sequela of acute pericarditis that impacts significantly on quality of life.^1^, ^2^, ^3^ IRP management remains challenging, with no US guidelines currently in place to aid clinicians and, although studies have demonstrated that corticosteroids are associated with increased recurrence risk, their use in IRP remains widespread.^1^, ^2^, ^3^, ^4^ Consequently, there is a need to understand the mechanisms driving IRP, to guide management that minimizes treatment‐associated harm.

A leading hypothesis is that IRP is mediated by autoinflammation.^1^ Autoinflammation is defined as excessive activity of the innate immune system, in the absence of an extrinsic stressor or autoreactive antibodies or T cells.^5^ It is best understood in the context of the monogenic systemic autoinflammatory disease (SAID). The prototypic SAID is familial Mediterranean fever (FMF), an autosomal recessive disease caused by mutations in the *MEFV* gene that result in overactivity of the pyrin inflammasome and interleukin (IL)‐1β secretion, which presents with episodic serositis.^5^

FMF prognosis has been transformed by prophylactic use of colchicine, an ancient drug that acts on the cytoskeleton, with pleiotropic effects including inhibition of inflammasome formation.^6^, ^7^ More recently, biologic anti‐IL‐1 agents, including anakinra and rilonacept, have revolutionized the management of colchicine‐refractory FMF and 3 other monogenic SAIDs: cryopyrin‐associated periodic syndrome (CAPS), TNF receptor–associated periodic syndrome (TRAPS), and mevalonate kinase deficiency (MKD).^6^, ^8^ These developments have highlighted the central role of IL‐1 in autoinflammation and provided a paradigm for the targeted treatment of relapsing inflammatory diseases. Importantly, randomized controlled trials have shown that these same agents are effective in IRP (Table 1).^9^, ^10^, ^11^, ^12^, ^13^ This was reflected both in the inclusion of colchicine and anakinra in the 2015 European Society of Cardiology (ESC) guidelines and the recent US Food and Drug Administration licensing of rilonacept and, interestingly, suggests that IRP may be mediated by autoinflammatory pathways.^14^

**Table 1:** Summary of Randomized Controlled Trials of Interventions in Recurrent Pericarditis

	Colchicine			IL‐1 receptor antagonist	Anti‐IL‐1 trap*
	CORE^9^	CORP^10^	CORP‐2^11^	Anakinra: AIRTRIP^12^	Rilonacept: RHAPSODY^13^
Design	RCT Open label, multicenter	RCT Double blind, multicenter	RCT Double blind, multicenter	Randomized withdrawal trial^*^ Double‐blind, multicenter	Randomized withdrawal trial^*^ Double‐blind, multicenter
Inclusion criteria	1st recurrence	1st recurrence	≥2nd recurrence	≥4th recurrence, CRP≥10 mg/L Corticosteroid dependent	≥2nd recurrence, CRP≥10 mg/L No recent DMARD use
Pericarditis cause	IRP (83%), autoimmune or postpericardiotomy (17%)	IRP (82%), autoimmune or postpericardiotomy (18%)	IRP (83%), post cardiac injury (9%), autoimmune (7%)	IRP (100%)	IRP (85%), Dressler’s or post pericardiotomy (15%)
Randomization criteria	N/A	N/A	N/A	Complete response and stopped NSAIDs, DMARD, and corticosteroids at 60 d^†^	Complete response and stopped NSAIDs, colchicine, and corticosteroids at 12 weeks^†^
Patient number	84 randomized 42 colchicine 42 nil	120 randomized 60 colchicine 60 placebo	240 randomized 120 colchicine 120 placebo	21 included, 21 randomized^†^ 11 anakinra 10 placebo	86 included, 61 randomized^†^ 30 rilonacept 31 placebo
Intervention group regimen	Colchicine 6 mo 1–2 mg/24 h loading on D0 0.5–1 mg/24 h thereafter	Colchicine 6 mo 1–2 mg/24 h loading on D0 0.5–1 mg/24 h thereafter	Colchicine 6 mo 0.5–1 mg/24 h	Anakinra (study duration)^‡^ 100 mg/kg per d	Rilonacept (study duration)^‡^ 320 mg loading dose 160 mg weekly thereafter
Control group regimen	No placebo	Placebo	Placebo	As per intervention group for 60 d then placebo^†^	As per intervention group for 12 weeks then placebo^†^
Concomitant medications	NSAID or prednisolone (≤4 wks then tapered)	NSAID or prednisolone (≤4 wks then tapered)	NSAID or prednisolone (≤4 wks then tapered)	Colchicine may be prescribed at discretion of physician	None
Primary end point(s)	Recurrence rate at 18 mo 24% vs 51%, *P*=0.02	Recurrence rate at 18 mo 24% vs 55%, *P*<0.01	Recurrence rate at 6 mo 22% vs 42%, *P*<0.01	Recurrence (%) at 8 mo 18.2% vs 90%, *P*=0.001 Median time to recurrence NE vs 72 d, *P*<0.001	Median time to recurrence NE vs 8.6 wks, *P*<0.001
Adverse events	No significant difference vs control group (14% vs 7%)	No significant difference vs control group (7% vs 7%) GI intolerance (7% vs 5%)	No significant difference vs control group (10% vs 10%) GI intolerance (7.5% vs 7.5%)	Injection site reactions (95%) Transient elevated transaminases (14.3%)	Injection site reactions (34%) URTI (mild/moderate) (23%)
Definition of recurrence	Pain and 1 of: fever, rub, ECG changes, effusion,^§^ raised WCC/ESR/CRP	Pain and 1 of: fever, rub, ECG changes, effusion,^§^ raised WCC/ESR/CRP	Pain and 1 of: fever, rub, ECG changes, effusion,^§^ raised WCC/ESR/CRP	Pain and raised CRP^‖^ and 1 of: fever, rub, ECG changes or effusion^§^	Pain and raised CRP^‖^ and 1 of: rub, ECG changes or effusion^§^

CRP indicates C‐reactive protein; DMARD: disease modifying antirheumatic drug; ESR, erythrocyte sedimentation rate; GI, gastrointestinal; IRP, idiopathic recurrent pericarditis; N/A, not applicable; NE, could not be estimated; NSAID, nonsteroidal anti‐inflammatory; RCT, randomized controlled trial; URTI, upper respiratory tract infection; and WCC, white cell count.

* Two further randomized controlled trials of the novel anti‐IL‐1 trap RPH‐104 are currently registered on clinical trials.org (NCT04692766, NCT05107934).

^†^ In randomized controlled trials, patients undergo an open label run‐in period on anakinra/rilonacept, following which responders are randomized to either the intervention or placebo group. Patients randomized to placebo who experience a disease recurrence are then offered bailout anakinra/rilonacept.

^‡^ Note pediatric dose adjustments for anakinra (2 mg/kg per 24 h) and rilonacept (4.4 mg/kg loading, 2.2 mg/kg maintenance).

^§^ New or worsening pericardial effusion.

^||^ For AIRTRIP, details of pain and CRP threshold values are not published; for RHAPSODY pain is defined as pain ≥2 using a numerical scale 0 to 10 and CRP ≥10 mg/L.

Despite the similarities between SAID and IRP, it is yet to be established whether clinical features or genetic determinants of autoinflammation are seen in IRP, and studies evaluating pericardial involvement in IL‐1‐mediated SAID are lacking. Here, we begin by characterizing the phenotype of 136 patients with IRP and analyze clinical features that are associated with complicated disease. We then conduct the first systematic assessment of pericarditis across IL‐1‐mediated SAIDs. We conclude with analysis of the 4 genes that cause these conditions in 128 patients with IRP, to identify whether the presence of variants in 1 or more of these genes is associated with an increased risk of IRP. Together these approaches evaluate the extent to which clinical features and genetic determinants of autoinflammation characterize IRP.

## Methods

The data underlying this article will be shared upon reasonable request to the corresponding author. This study complies with the Declaration of Helsinki. Informed written consent was provided by all study participants and ethical approval was obtained from the Royal Free Hospital and University College Medical School Research Ethics Committee (REC reference number 06/Q0501/42).

### Patient Ascertainment

This study included all consecutive patients with a diagnosis of IRP (n=136) or IL‐1‐mediated monogenic SAIDs (n=1910) that were reviewed between October 17, 2000 and March 25, 2021 in a single UK reference center (CAPS and autoinflammatory disease treatment service, National Amyloidosis Centre, Royal Free Hospital & UCL Division of Medicine, London). Data pertaining to demographics, comorbidity, clinical presentation, complications, and treatment were gathered from electronic medical records. Participant ethnicity was self‐reported and participant ancestry was categorized based on self‐reported descent according to groups defined in the Genome Aggregation Database (gnomAD, v2.1.1), thereby facilitating comparison of minor allele frequency with ancestry‐matched populations. For patients with monogenic SAIDs, case notes were reviewed for a history of acute or recurrent pericarditis that met ESC diagnostic criteria.^14^ Collection of data from medical records and its analysis was performed from April 1 to August 3, 2021.

### DNA Extraction and Sequencing

Genomic DNA (n=128) was extracted from whole blood, as previously described, or saliva, collected in OraGENE kits, according to the manufacturer’s protocol, and was stored for future sequencing.^15^ Next‐generation sequencing was performed using one of two custom Illumina panels (TruSeq panel, n=44; AmpliSeq panel, n=84) that include the four genes: *MEFV, MVK, NLRP3, and TNFRSF1A*. Full details of the genomic regions covered by each panel are available in Data S1. Next‐generation sequencing libraries were prepared using the TruSeq or AmpliSeq Custom Amplicon Method (Illumina). All sequencing was performed on the Illumina MiSeq platform between February 8, 2018 and May 24, 2021.

### Variant Calling and Annotation

Reads were aligned to the human reference genome (hg19) and variants were called using Somatic Variant Caller (Illumina). Variants identified for each patient were filtered and prioritized using Variant Interpreter Software (Illumina). The analysis of germline alleles (defined as an alternative allele found in ≥35% reads) and somatic variants (defined as an alternative allele seen in 5%–34% reads) was restricted to regions covered at >50× and >200× depth, respectively.

To identify candidate variants, we first selected nonsynonymous and splice site variants. These were then cross‐referenced against the databases ClinVar (https://www.ncbi.nlm.nih.gov/clinvar/; accessed [19/07/21]*)* and InFevers (https://infevers.umai‐montpellier.fr/; accessed [19/07/21]*)* to identify known pathogenic or likely pathogenic alleles.^16^, ^17^, ^18^, ^19^, ^20^ In parallel, variants were annotated according to the minor allele frequency reported on the gnomAD for the corresponding ancestry group. Rare variants (minor allele frequency <0.01) were then subjected to in silico pathogenicity prediction using the Combined Annotation Depletion Dependent tool.^21^, ^22^ A scaled Combined Annotation Depletion Dependent score >10 was considered evidence of deleterious potential.^22^

### Rare Variant Analysis by Burden Association Testing

Burden association tests were performed when multiple rare deleterious variants (minor allele frequency <0.01, Combined Annotation Depletion Dependent score >10) were identified in a single gene, by comparing the combined frequency of all rare deleterious variants in cases to that of ancestry‐matched controls. Individuals with exome sequencing data available in gnomAD v2.1.1 were used as reference controls. To confirm that cases and controls were appropriately matched, we also undertook a burden association test comparing the combined frequency of synonymous rare variants between the 2 groups. Genetic analysis was performed between June 4 and August 3, 2021.

### Statistical Analysis

Numerical variables are presented as median ± interquartile range, while categorical data are described as absolute counts and percentages. Differences between groups were assessed with the Mann–Whitney *U* test for continuous data and the χ^2^ or Fisher exact test for discrete data. Where multiple independent variables were found to be associated with the response variable, these were included in a multiple logistic regression model as explanatory variables, together with sex and age of onset, to account for any confounding effects. All statistical analyses and visualization of data were conducted in R statistics (v4.0.3, https://www.R‐project.org *)*. For multiple logistic regression analyses, the adjusted odds ratio (aOR), 95% CI, and *P* value are reported. *P* values <0.05 are considered statistically significant.

## Results

### Demographic and Clinical Features of Patients With IRP

We characterized the clinical features of our cohort of 136 patients diagnosed with IRP (Table 2). IRP showed an even sex distribution with a median age of onset of 31 years, and the majority of patients had no comorbidities (83/136, 61.0%). The disease was sporadic in most cases, with family history in a first‐degree relative reported in only 6 patients (4.4%). Most affected individuals showed evidence of extrapericardial inflammation during disease flares and, notably, 68/136 (50%) had a history of extrapericardial effusion (pleural effusion and/or ascites). Median follow‐up from first to last study visit was 12 months (interquartile range, 5–37 months) and the median time from first pericarditis diagnosis to first visit date was 4 years (interquartile range, 1–7 years) (Figure S1).

**Table 2:** Demographics and Clinical Features of Patients With IRP

	All patients with IRP (n=136)
Demographics	
Sex (female), n (%)	68 (50.0)
Onset, median [IQR]	31.00 [23.0–40.0]
Patient reported ethnicity, n (%)*	
Asian or Asian British	5 (3.7)
Black or Black British	12 (8.8)
Mixed ethnicity	3 (2.2)
Other ethnicity (Arab)	6 (4.4)
Unknown or not disclosed	1 (0.7)
White or White British	109 (80.1)
Family history, n (%)^†^	6 (4.4)
Year of first confirmed episode of acute pericarditis, n (%)	
1991–2015	90 (66.2)
2016–2020	46 (33.8)
Comorbidities, n (%)	
No comorbidity	83 (61.0)
One or more comorbidity^‡^	53 (39.0)
Hypertension	11 (8.1)
Osteoporosis or osteopenia	9 (6.6)
Supraventricular arrhythmia	8 (5.9)
Thyroid disease	7 (5.1)
Type 2 diabetes	5 (3.7)
Depression	5 (3.7)
Asthma	4 (2.9)
Previous malignancy	4 (2.9)
Renal disease	4 (2.9)
Endometriosis	3 (2.2)
Clinical features of acute pericarditis episodes, n (%)	
Chest pain	136 (100.0)
Elevated C‐reactive protein	121 (89.0)
Fever >39°C	72 (52.9)
Pericardial effusion	110 (80.9)
Pleural effusion	68 (50.0)
Ascites	11 (8.1)
Arthralgia	41 (30.1)
Rash	15 (11.0)
Complications, n (%)^§^	
Cardiac tamponade	27 (19.9)
Myocardial involvement	15 (11.0)
Constriction	1 (0.7)
Corticosteroid dependence	39 (28.7)
Chronic chest pain	51 (37.5)
Chronic fatigue	19 (14.0)
Management^‖^	
Number of disease‐modifying drugs tried, median [IQR, range]	2 [1–3, 0–7]
No treatment or simple analgesia only, n (%)	10 (7.4)
Colchicine, n (%)	125 (91.9)
Corticosteroid, n (%)	77 (56.6)
Anakinra, n (%)	19 (14.0)
Disease‐modifying antirheumatic drug(s), n (%)	27 (19.9)
Other biologic drug, n (%)	3 (2.2)
Pericardiectomy	1 (0.7)
Treatment of presenting episode, n (%)^‖^	
No treatment or simple analgesia only	84 (61.8)
Colchicine only	26 (19.1)
Corticosteroid only	21 (15.4)
Colchicine and corticosteroid	5 (3.7)

IQR indicates interquartile range; and IRP, idiopathic recurrent pericarditis.

* Self‐reported ethnicity using questionnaire that included ethnicity categories recommended for use in England and Wales.

^†^ First‐degree relative with 1 or more episode(s) of confirmed acute pericarditis.

^‡^ Specific diagnoses are reported where a diagnosis was observed in 3 or more patients.

^§^ Myocardial involvement is defined as a raised Troponin I/T, or myocardial inflammation on cardiac magnetic resonance imaging (MRI). Corticosteroid dependence is defined as requiring continuous daily corticosteroids for 6 months or longer. Chronic chest pain is defined as chest pain in the presence of normal investigations (inflammatory markers±ECG and imaging (echocardiogram and/or MRI)) on 2 or more occasions. Chronic fatigue is defined as patient‐reported fatigue between disease flares.

^‖^ Disease‐modifying drugs comprise colchicine, corticosteroid, anakinra, disease‐modifying antirheumatic drug, or other biologic drug. Simple analgesia includes nonsteroidal anti‐inflammatory drugs.

### Management of Patients With IRP

**Figure 1:**
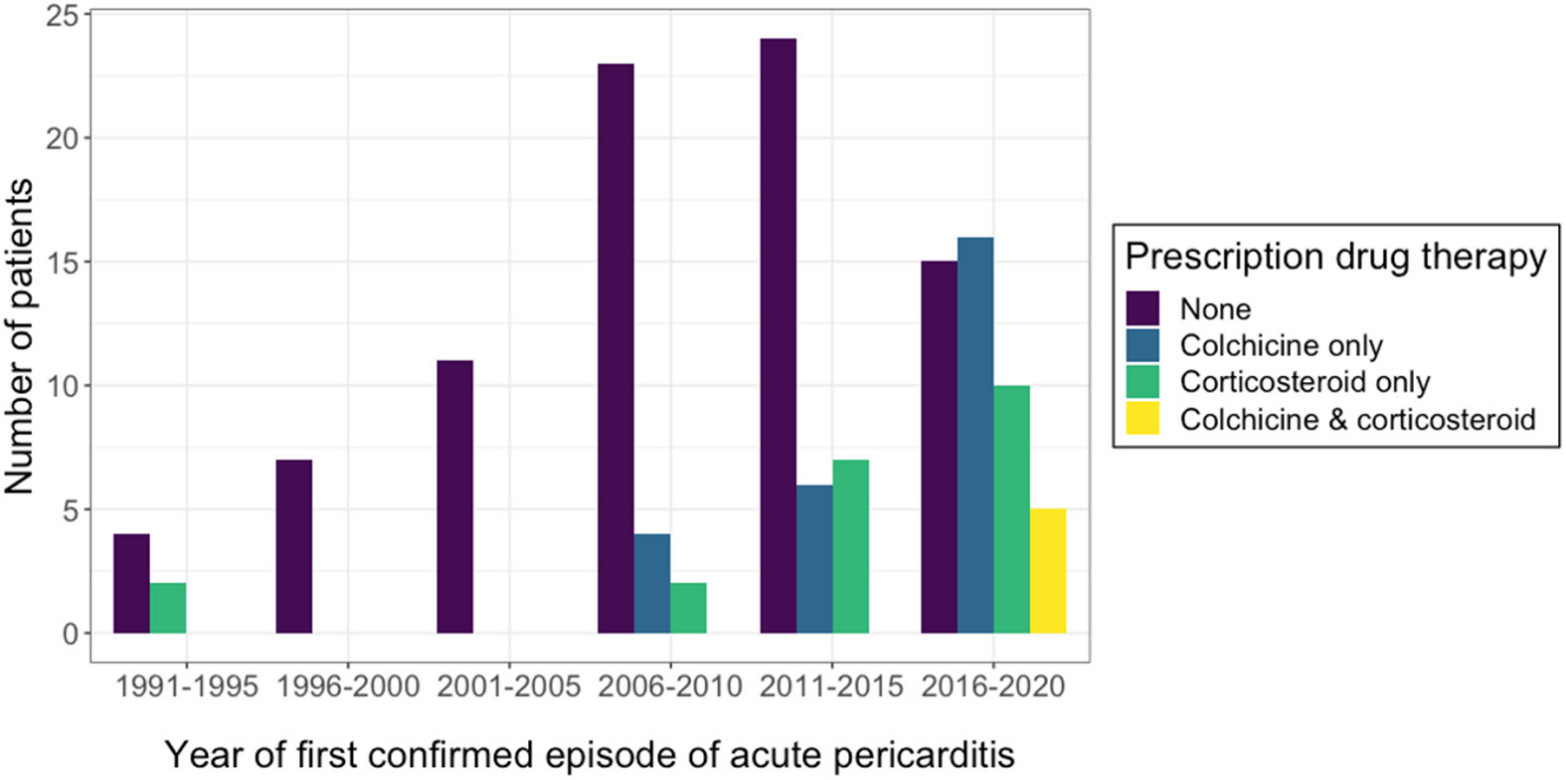
Management of the first confirmed episode of acute pericarditis in patients with IRP. Bar graph showing the number of patients managed with each prescription agent stratified by the year of the first confirmed episode of acute pericarditis. Management refers to prescription medications and does not include medications available over‐the‐counter, such as nonsteroidal anti‐inflammatory drugs. IRP indicates idiopathic recurrent pericarditis.

Treatment details are summarized in Table 2. Colchicine was the most commonly prescribed drug, used in 125/136 patients (91.9%), followed by corticosteroids, in 77/136 (56.6%). Of note, colchicine use at first pericarditis diagnosis was low (31/136, 22.8%), though it was significantly higher in patients diagnosed from 2016, after publication of the 2015 ESC guidelines, compared with those presenting up to and including 2015 (21/46 versus 10/90, *P*<0.0001) (Figure 1). Corticosteroids were used in the presenting episode in 26/136 cases (19.1%) and more frequently from 2016 onwards (15/46 versus 11/90, *P*=0.006).

Of the 19/136 individuals (14.0%) who were treated with the recombinant IL‐1 receptor antagonist anakinra, 18 received 100 mg daily as prophylaxis. Among those treated with prophylactic anakinra, only 1 episode of acute pericarditis was recorded over a median follow‐up period of 24 months. In the affected patient, no further episodes were seen after dose escalation to 200 mg daily.

### Factors Associated With Complications in IRP

**Figure 2:**
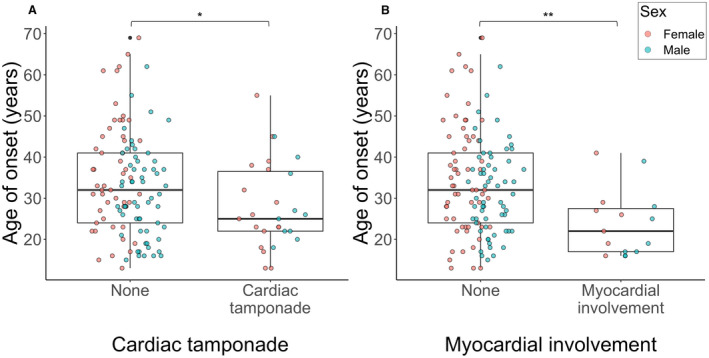
Association between acute cardiac complications and age of onset. Box plots showing the median age of onset in patients with or without a history of (**A**) cardiac tamponade and (**B**) myocardial involvement. Comparison is made by a multiple logistic regression analysis (Tables S1 and S2). *P<0.05; **P<0.01.

Acute cardiac complications occurred in a significant minority of patients. Cardiac tamponade, necessitating emergency pericardiocentesis, was reported in 27/136 cases (19.9%) and myocardial involvement, defined as a raised troponin and/or evidence of myocardial inflammation on cardiac MRI, in 15/136 cases (11.0%). Cardiac tamponade was reported during a single episode of acute pericarditis in 26 patients, and in 2 discrete episodes in the remaining individual. Cardiac tamponade was associated with earlier disease onset, although the effect size was small (aOR, 0.96 [95% CI, 0.92–0.99], *P*=0.028) (Figure 2A, Table S1). Importantly, we identified no association between a history of cardiac tamponade and the development of the common chronic complications of corticosteroid dependence, chronic chest pain or chronic fatigue. Myocardial involvement was also associated with an earlier age of onset (aOR, 0.91 [CI, 0.85–0.97], *P*=0.0063) (Figure 2B, Table S2).

Chronic complications were more common. The most frequent was chronic chest pain, defined as pericarditic chest pain with normal inflammatory markers and without findings consistent with acute pericarditis on examination or investigation on 2 or more occasions, which was reported in 51/136 patients (37.5%).

Corticosteroid dependence, defined as continuous daily corticosteroid use at doses greater than prednisolone 5 mg (or equivalent) for 6 months or longer, was also common, occurring in 39/136 patients (28.7%). A significant minority of corticosteroid‐dependent patients developed physical health problems suggestive of corticosteroid toxicity (11/39, 28.2%). Of particular note, 9/39 (23.1%) were diagnosed with osteoporosis or osteopenia, accounting for all cases of these diagnoses in the entire IRP cohort, and 1 patient developed adrenal insufficiency requiring lifelong glucocorticoid replacement. We also observed 1 new diagnosis of hypertension, while 1 patient with pre‐existing type 2 diabetes and hypertension required escalation of antihypertensive therapy and the initiation of insulin.

Chronic fatigue, defined as patient‐reported fatigue between flares, was reported by 19/136 patients (14.0%). All patients with chronic fatigue also reported chronic chest pain.

Patients with a history of corticosteroid dependence were more likely to report chronic pain (aOR, 2.8 [95% CI, 1.3–6.5], *P*=0.012) or chronic fatigue (aOR, 4.8 [95% CI, 1.6–14], *P*=0.0041) than those without (Tables S3 and S4). Those with a history of myocardial involvement were less likely to report chronic pain (aOR, 0.12 [95% CI, 0.0054–0.55], *P*=0.049) (Table S3). Corticosteroid dependence was also associated with a history of extrapericardial effusion (aOR, 2.6 [95% CI, 1.2–6.3], *P*=0.023) (Table S5).

Taken together, these clinical data illustrate the frequent problem of corticosteroid dependence among this patient group and suggest that this may be associated with increased risk of chronic symptoms.

### Pericarditis in Monogenic SAIDs: Prevalence and Response to Treatment

Pericarditis is often described as a feature of autoinflammatory diseases, but no previous study has compared its prevalence across the 4 IL‐1‐mediated monogenic SAIDs. In our cohort of 1910 individuals, we observe pericarditis in each of these conditions, and find that it is most prevalent in MKD (Table 3). At diagnosis, all patients had marked systemic involvement and no patient reported pericardial chest pain as their first symptom. Cardiac tamponade necessitating pericardiocentesis was only observed in one patient with MKD and no cases of pericarditis complicated by myocardial involvement were recorded.

**Table 3:** Prevalence of Acute and Recurrent Pericarditis in Monogenic Systemic Autoimflammatory Diseases

Disease (causative gene, inheritance)	History of pericarditis	
	Acute	Recurrent
Mevalonate kinase deficiency (MKD) (*MVK*, recessive)	3.7% (3/82)	2.4% (2/82)
Cryopyrin‐associated periodic fever syndrome (CAPS) (*NLRP3*, dominant)	1.3% (3/234)	0.4% (1/234)
Familial Mediterranean fever (FMF) (*MEFV*, recessive)	0.4% (5/1316)	0.1% (1/1316)
TNF receptor‐associated periodic fever syndrome (TRAPS) (*TNFRSF1A*, dominant)	0.7% (2/278)	0.0% (0/278)

Of the 17 confirmed episodes of pericarditis, 14 (82.4%) occurred before establishment on prophylactic immunomodulatory therapy, 2 while on low‐dose colchicine, and 1 while on the anti‐IL‐1β monoclonal antibody canakinumab. Given the very low incidence of pericarditis in the general population, with population estimates ranging from 3.3 to 27.7 per 100 000 person‐years, our data demonstrate that pericarditis is a definite but uncommon feature of SAID, which typically occurs before initiation of prophylactic immunomodulatory treatment.^23^, ^24^

### Candidate Gene Analysis of *MEFV, MVK, NLRP3*, and *TNFRSF1A* in IRP Cases

Having shown that pericarditis is a feature of FMF, MKD, CAPS and TRAPS, we next assessed whether variants in the genes responsible for these conditions are also observed in patients with IRP. *MEFV, MVK, NLRP3*, and *TNFRSF1A* were sequenced in 128 patients with IRP, with a mean coverage of 1484×. More than 99% of bases had been sequenced at a depth sufficient to detect germline mutations (>50×). Moreover, 97% of nucleotides were sequenced at >200× depth, providing sufficient coverage to detect somatic mutations, which is of interest given that somatic mutations are implicated in late‐onset autoinflammatory diseases.^25^, ^26^

We first considered germline mutations that had previously been classified as pathogenic or likely pathogenic by consensus agreement.^27^ We found no such variants in *MVK, NLRP3*, or *TNFRSF1A*. Conversely, 5 patients with IRP (5/128, 3.9%) harbored pathogenic or likely pathogenic variants in the *MEFV* gene (Table 4). However, all carried a single heterozygous mutation, and therefore did not meet consensus criteria for a genetic diagnosis of FMF, which is an autosomal recessive condition.^27^

**Table 4:** Pathogenic or Likely Pathogenic Variants Identified in Idiopathic Recurrent Pericarditis Cases

Gene	Variant		Consensus classification	Patient ID	Patient ancestry	Ancestry‐matched MAF
	HGVSc	HGVSp				
*MEFV*	c.2080A>G	p.(Met694Val)	Pathogenic	1	NFE	0.0005
				2	NFE	0.0005
	c.2084A>G	p.(Lys695Arg)	Likely pathogenic	3	NFE	0.0068
				4	NFE	0.0068
	c.2177T>C	p.(Val726Ala)	Pathogenic	5	Ashkenazi Jewish	0.0398

Variants that have been classified as pathogenic or likely pathogenic by international consensus that were identified on sequencing of 4 candidate genes *(MEFV, MVK, NLRP3, TNFRSF1A)* in 128 IRP cases.

HGVS indicates Human Genome Variation Society; HGVSc, HGVS coding sequence; HGVSp, HGVS protein sequence; MAF, minor allele frequency; *MEFV*, MEFV innate immunity regulator, pyrin; and NFE, non‐Finnish European.

Given that the R121Q (previously known as R92Q*) TNFRSF1A* variant had previously been reported in patients with IRP of European ancestry, we next investigated whether it was associated with the disease in our cohort.^28^ This variant is classified as a variant of unknown significance in TRAPS by consensus agreement.^27^ We found that the allele frequency of R121Q among our 100 patients of non‐Finnish European ancestry (5/200, 2.5%) and that described by Cantarini et al (6/262, 2.3%) were not significantly different from that observed among ancestry‐matched healthy controls in the gnomAD database (2564/129 036, 2.0%) (*P*=0.60 and 0.66, respectively).^28^ This argues against the notion that R121Q has a pathogenic role in IRP.

To determine whether previously unreported variants might be implicated in IRP, we next looked for rare variants predicted to be deleterious by in silico tools. While no such changes were seen in *TNFRSF1A*, a total of 8 rare deleterious variants were identified in the other 3 genes in 11 individuals (Table 5). Of note, 9 individuals (9/128, 7.0%) carried a rare deleterious variant in the *MEFV* gene, meaning that 7.8% of patients with IRP (10/128) carried a variant in *MEFV* that was either known or predicted to be damaging. Of note, all patients with IRP with a candidate *MEFV* variant had raised inflammatory markers during attacks and none had evidence of myocardial involvement (Table S6).

**Table 5:** Rare Deleterious Variants Identified in Idiopathic Recurrent Pericarditis Cases

Gene	Variant		In silico predication	Patient ID	Patient ancestry	Ancestry‐matched MAF
	HGVSc	HGVSp				
*MEFV*	c.26T>A	p.(Leu9Gln)	Damaging	6	NFE	Novel
	c.124C>T	p.(Arg42Trp)	Damaging	7	NFE	1.76 10^−5^
	c.1337A>C	p.(Glu446Ala)	Damaging	8	NFE	7.74 10^−5^
				9	NFE	7.74 10^−5^
	c.1759+1G>A	…	Damaging	10	NFE	Novel
	c.2080A>G	p.(Met694Val)	Damaging	1	NFE	0.0005
				2	NFE	0.0005
	c.2084A>G	p.(Lys695Arg)	Damaging	3	NFE	0.0068
				4	NFE	0.0068
*MVK*	c.677+7delT	…	Damaging	11	NFE	Novel
*NLRP3*	c.2711C>T	p.(Ser906Leu)	Damaging	12	NFE	1.76 10^−5^

Rare deleterious variants (ancestry matched minor allele frequency <1% that are predicted in silico to be damaging [scaled CADD score >10]) identified on sequencing of 4 candidate genes *(MEFV, MVK, NLRP3, TNFRSF1A*) in 128 IRP cases.

HGVS indicates Human Genome Variation Society; HGVSc, HGVS coding sequence; HGVSp, HGVS protein sequence; MAF, minor allele frequency; *MEFV*, MEFV innate immunity regulator, pyrin; NFE, non‐Finnish European; *MVK*, mevalonate kinase; and *NLRP3*, NLR family pyrin domain containing 3.

To further investigate the pathogenic significance of rare deleterious variants in *MEFV*, we compared their combined frequency in patients with IRP who were of non‐Finnish European ancestry (hereafter referred to as European ancestry) to that of all rare deleterious variants in *MEFV* in ancestry‐matched healthy controls in the gnomAD database. In this subgroup of 100 patients, we observe rare variants in *MEFV* that are predicted to be damaging in patients with IRP more frequently than expected (allele frequency 9/200 versus 2932/12 9200, *P*=0.040) (Table 6). Importantly, the combined frequencies of rare synonymous *MEFV* variants were comparable in cases and controls, confirming that the 2 groups were appropriately matched (Table S7).

**Table 6:** Burden Association Test of Rare Deleterious Variants in *MEFV* in Individuals of European Descent With IRP

Gene	Combined frequency of rare deleterious variants		*P* value
	Allele frequency in cases	Allele frequency in controls	
*MEFV*	9/200 (4.5%)	2932/129200 (2.3%)	0.040^*^

Combined allele frequency of rare deleterious variants (number of variants divided by the total number of alleles) among the 100 NFE IRP cases and the 64 600 ancestry‐matched controls taken from the gnomAD database; comparison in made using a 1‐sided Fisher test. *P<0.05.

gnomAD indicates genome aggregation database; IRP, idiopathic recurrent pericarditis; *MEFV*, MEFV innate immunity regulator, pyrin; and NFE, non‐Finnish European.

No somatic variants were detected in any of the 4 examined genes.

## Discussion

IRP is widely regarded as a single system cardiac disease, and current ESC guidelines for the diagnosis of IRP and for recognition of disease recurrence focus solely on pericardial inflammation. However, our findings illustrate that systemic inflammation and localized extrapericardial inflammation, most notably serositis, are common features of the disease, reframing IRP within the spectrum of systemic inflammatory diseases.^14^ Indeed, we observe an association between the presence of extrapericardial effusion(s) and corticosteroid dependence, suggesting that a more comprehensive approach is indicated when assessing disease activity.

While corticosteroid dependence is known to carry serious risks of complications and impact significantly on patient quality of life, corticosteroids remain the second‐line treatment recommendation in European guidelines after low‐dose colchicine.^2^, ^14^, ^29^ Notably, the second most common comorbidity among all patients with IRP in this cohort was iatrogenic osteoporosis or osteopenia. Moreover, in our cohort, a history of corticosteroid dependence was associated with chronic chest pain and fatigue. There is a complete lack of data on whether pharmacological treatment of symptoms, in the absence of objective evidence of inflammation, is beneficial, because such cases have been excluded from trials (Table 1). Indeed, the association we observe between corticosteroid dependence and chronic pain may be, in part, driven by the inappropriate treatment of noninflammatory symptoms. This, together with the low early uptake of colchicine in our cohort, with less than half of those presenting with acute pericarditis after publication of the 2015 ESC guidelines receiving colchicine at diagnosis, a finding replicated in a recent US study, further highlight the current barriers to adoption of evidence‐based treatment.^3^ Accordingly, there is a need to understand the mechanisms underlying both the disease and its complications to guide earlier use of targeted interventions, thereby minimizing iatrogenic harm and reducing the risk of chronic sequelae.

In this study we approach this by analyzing 4 candidate genes, in which pathogenic mutations are known to cause IL‐1‐mediated autoinflammatory disease. Importantly, we first establish that IRP represents a distinct disease with no patients in our cohort meeting consensus criteria for a genetic diagnosis of FMF, MKD, CAPS, or TRAPS.^27^ We also did not find any association between 3 of these genes (*MVK, NLRP3*, and *TNFRSF1A*) and IRP. This is of particular note in the case of *NLRP3* in view of a recent study that showed upregulation of NLRP3 inflammasome activity in the pericardia of patients with recurrent pericarditis complicated by constriction.^30^ However, our genetic analysis did find that 7.8% of patients with IRP (10/128) harbor variants in *MEFV* that are either known or predicted to be pathogenic, and we find an association between rare deleterious variants in this gene and IRP, suggesting that the presence of heterozygous variants in *MEFV* may be associated with an increased risk of the disease. While FMF is generally an autosomal recessive disease, asymptomatic carriers of a single pathogenic *MEFV* variant have elevated inflammatory markers compared with wild‐type controls.^31^ More recent functional work has also demonstrated that neutrophils from asymptomatic *MEFV M694V* heterozygotes show increased pro‐inflammatory activity.^32^ These findings indicate a gene–dosage effect, whereby gain‐of‐function mutations in *MEFV* result in increased innate immune activity. Accordingly, our findings suggest that the presence of a single *MEFV* mutation may predispose carriers to the development of recurrent pericarditis.

While these monogenic SAIDs are often said to manifest with pericarditis, the prevalence of pericarditis has only previously been investigated in FMF.^33^ In our cohort, we observe pericarditis in all 4 diseases examined. We also demonstrate that pericarditis typically occurs before establishment on prophylactic therapy targeting the IL‐1 axis. Complete disease control using these agents is now widely achieved in these conditions, and this may both result in an underestimate of the natural incidence of pericarditis in SAIDs in our study while also providing a paradigm for how the treatment of IRP might be approached.^6^

The concept of treat‐to‐target is widely used in rheumatology and in the management of SAIDs, with the aim of obtaining optimal disease responses with minimal treatment‐associated morbidity.^34^ The approach involves the identification of a set target at a defined treatment interval, with a stepwise plan for escalation should the treatment target not be reached. This has clear advantages in standardizing treatment while also facilitating shared decision making with patients. Importantly, this strategy has been shown to improve remission rates in randomized controlled trials and is the recommended treatment approach in the 4 monogenic SAIDs included in this study.^34^, ^35^ For example, in FMF, consensus guidance recommends gradual escalation of colchicine dose, routinely up to 3 mg/d in adults, guided by treatment response, and, in patients truly refractory to higher doses of colchicine, anti‐IL‐1 agents are then added to treatment.^7^ Recognition of IRP as falling within the spectrum of SAID should facilitate a culture change away from overreliance on corticosteroids, and their associated harms, to a more consistent, targeted approach.

Importantly though, the treat‐to‐target strategy relies on validated measures of disease activity, an area that has been neglected in research into IRP. Indeed, the difficulty of distinguishing disease activity from disease damage in chronic diseases, and specifically in autoinflammatory diseases, is well recognized.^36^ In previous studies of IRP, the number or timing of recurrences has been widely used. However, in IRP there is significant heterogeneity in the presentation of disease recurrence, both between and within patients, and in the definitions used by studies, which limits the generalizability and reproducibility of such research (Table 1). This is particularly pertinent given that more than one third of our patients report chest pain without evidence of acute pericardial inflammation on investigation. Consensus guidance on the management of SAID advocates the parallel use of disease activity and disease damage scores, to facilitate titration of pharmacological interventions against disease activity, while also monitoring for complications that may necessitate alternative treatment approaches and for which the benefit of immunomodulatory drugs may not have been proven.^6^, ^7^, ^8^ Given the adverse outcomes associated with prolonged corticosteroid use and both the potential side effects and cost of newer biologic agents, distinguishing between active inflammatory disease amenable to immunomodulation and established noninflammatory complications is crucial to minimize treatment‐associated harms. The development and validation of disease activity and damage scores are therefore urgently needed to both improve patient care through unbiased disease monitoring and to facilitate robust, reproducible research into this condition.

Our study carries a number of limitations. First, the retrospective design means our analysis will not capture features that were not documented, reported, or investigated contemporaneously. As such there is a risk of information bias, and, accordingly, prospective studies of pericarditis are much needed. Additionally, our IRP cohort was recruited through a national referral center, which has likely resulted in an enrichment of more severe phenotypes in our cohort, and there is an outstanding need for collection of data in large, unselected cohorts and, importantly, in patients of diverse ethnicities. Finally, given the observational design of the study, we are not able to identify causal relationships.

For our genetic analysis, we utilize publicly available controls, listed in the gnomAD database, meaning that sequencing was performed on different platforms and that detailed information about control phenotypes was not available. To assess the possibility of mismatching between cases and controls, we conducted a parallel analysis of rare synonymous variants, which found no significant differences between the 2 groups, suggesting they are well matched (Table S4). Nonetheless, our findings will need to be validated in a second cohort, and, importantly, in patients of non‐European ancestries, which our study was underpowered to consider. Finally, the assessment of novel or uncategorized variants based on in silico pathogenicity prediction must be interpreted with caution, and functional testing of novel candidate variants is needed to establish their potential contribution to IRP.

## Conclusions

An increasing body of evidence, including our finding that damaging variants in *MEFV* may confer an increased risk of IRP, implicates autoinflammation and the IL‐1 pathway in the pathogenesis of this disease. Despite this, corticosteroid use remains widespread and dependence is associated with poor outcomes. Taken together, these data support reduced reliance on corticosteroids in IRP management and wider uptake of innate immune modulators targeted to inflammatory activity, while ongoing work continues to investigate the exact mechanisms by which autoinflammatory pathways drive this debilitating disease.

## Sources of Funding

This work is supported by a British Heart Foundation Clinical Research Training Fellowship (FS/19/67/34697).

## Disclosures

CP reports receiving speaker fees from Sobi. FC reports receiving funding and consultancy fees from Boehringer Ingelheim. HJL reports receiving consulting fees from Novartis and Sobi. The remaining authors have no disclosures to report.

## Supporting information

Data S1
Table S1–S7
Figure S1
