# Supplementary material for: Pericarditis and Autoinflammation: A Clinical and Genetic Analysis of Patients With Idiopathic Recurrent Pericarditis and Monogenic Autoinflammatory Diseases at a National Referral Center
Source: J Am Heart Assoc. 2022 Jun 6;11(11):e024931. doi: 10.1161/JAHA.121.024931 (PMC9238712; doi:10.1161/JAHA.121.024931)
Supplement: Supplementary file 1 — Data S1 Table S1–S7 Figure S1 [file JAH3-11-e024931-s001.pdf]

Supplemental Material

Data S1.

Supplemental Methods

Details of the custom next generation sequencing panels used

NGS was performed using a custom TruSeq panel (Illumina) (n=44) or custom AmpliSeq panel (Illumina) (n=84). The TruSeq panel consists of 377 amplicons covering a total of 49.275 kB. These comprise the coding regions and flanking intron sequences of 20 genes: *ADA2*, *CARD14*, *IL1RN*, *IL36RN*, *LPIN2*, *MEFV*, *MVK*, *NLRC4, NLRP12, NLRP3, NOD2, PLCG2, PSMB4, PSMB8, PSMB9, PSTPIP1, SH3BP2, TNFRSF1A, TMEM173 (STING), TNFAIP3*. For this panel, NGS libraries were prepared using the TruSeq Custom Amplicon method (Illumina). The AmpliSeq panel consists of 393 amplicons covering a total of 65.807 kB. These comprise the targets of the TruSeq panel and an additional four genes: *OTULIN, RBCK1, SLC29A3, UBA1*. For the AmpliSeq panel, NGS libraries were prepared using the AmpliSeq Custom Amplicon Method (Illumina). For both panels, sequencing was performed on the Illumina MiSeq platform.

Table S1. Multiple logistic regression model of cardiac tamponade.

	Reference level	***Adjusted OR (95% CI)***	***p value***
(Intercept)	-	1.44 (n/a)	n/a
Sex	Female	0.445 (0.177 – 1.07)	0.0752
Age of onset	-	0.956 (0.915 – 0.993)	0.0275*

*CI: confidence interval; OR: odds ratio. *p<0.05;**p<0.01;***p<0.001.*

Table S2. Multiple logistic regression model of myocardial involvement.

	Reference level	*Adjusted OR (95% CI)*	*p value*
(Intercept)	-	2.92 (n/a)	n/a
Sex	Female	0.846 (0.260 – 2.78)	0.779
Age of onset	-	0.911 (0.846 – 0.969)	0.00632**
Chronic pain	Absent	0.101 (0.00538 – 0.549)	0.0315*

*CI: confidence interval; OR: odds ratio. *p<0.05;**p<0.01;***p<0.001.*

Table S3. Multiple logistic regression model of chronic chest pain.

	Reference level	***Adjusted OR (95% CI)***	***p value***
(Intercept)	-	0.752 (n/a)	n/a
Sex	Female	0.575 (0.267 – 1.23)	0.153
Age of onset	-	0.996 (0.965 – 1.03)	0.794
History of corticosteroid dependence	Absent	2.82 (1.26 – 6.45)	0.0121*
History of myocardial involvement	Absent	0.120 (0.00639 – 0.672)	0.0489*

*CI: confidence interval; OR: odds ratio. *p<0.05;**p<0.01;***p<0.001.*

Table S4. Multiple logistic regression model of chronic fatigue.

	Reference level	*Adjusted OR (95% CI)*	*p value*
(Intercept)	-	0.101 (n/a)	n/a
Sex	Female	0.413 (0.121 – 1.24)	0.129
Age of onset	-	1.01 (0.965 – 1.05)	0.802
History of corticosteroid dependence	Absent	4.75 (1.66 – 14.4)	0.00414**

*CI: confidence interval; OR: odds ratio. *p<0.05;**p<0.01;***p<0.001.*

Table S5. Multiple logistic regression model of corticosteroid dependence.

	Reference level	*Adjusted OR (95% CI)*	*p value*
(Intercept)	-	0.0746 (n/a)	n/a
Sex	Female	0.530 (0.225 – 1.22)	0.139
Age of onset	-	1.03 (0.995 – 1.06)	0.104
Chronic chest pain	Absent	3.36 (1.49 – 7.80)	0.00402**
History of extra-pericardial effusion	Absent	2.64 (1.16-6.28)	0.0231* .

*CI: confidence interval; OR: odds ratio. *p<0.05;**p<0.01;***p<0.001.*

Table S6. Demographics and clinical features of patients with idiopathic recurrent pericarditis, stratified by *MEFV* genotype.

	Individuals with no variant in *MEFV*$^{\chi}$ (n=118)	Individuals with a variant in *MEFV*$^{\chi}$ (n=10)
**Demographics**		
Sex {female}, n (%)	56 (47.5)	7 (70.0)
Onset, median [IQR]	31.00 [23.0 – 39.0]	35.50 [26.0 – 40.5]
Ancestry, n (%)*		
African ancestry	10 (8.5)	0 (0.0)
Ashkenazi Jewish ancestry	4 (3.4)	1 (10.0)
Mixed ancestry	3 (2.5)	0 (0.0)
Non-Finnish European ancestry	91 (77.1)	9 (90.0)
Other ancestry	6 (5.1)	0 (0.0)
South Asian ancestry	3 (2.5)	0 (0.0)
Unknown or not disclosed	1 (0.8)	0 (0.0)
Family history, n (%)$^{\Psi}$	5 (4.2)	1 (10.0)
Year of first acute pericarditis diagnosis, n (%)		
1991 – 2015	76 (64.4)	8 (80.0)
2016 - 2020	42 (35.6)	2 (20.0)
**Clinical features of acute pericarditis episodes, n (%)**		
Chest pain	118 (100.0)	10 (100.0)
Elevated C-reactive protein	106 (89.8)	10 (100.0)
Fever over 39°C	63 (53.4)	4 (40.0)
Pericardial effusion	97 (82.2)	6 (60.0)
Pleural effusion	59 (50.0)	4 (40.0)
Ascites	11 (9.3)	0 (0.0)
Arthralgia	36 (30.5)	3 (30.0)
Rash	14 (11.9)	0 (0.0)
**Complications, n (%)**$^{\pm}$		
Cardiac tamponade	23 (19.5)	4 (40.0)
Myocardial involvement	14 (11.9)	0 (0.0)
Constriction	1 (0.8)	0 (0.0)
Corticosteroid dependence	35 (30.0)	2 (20.0)
Chronic chest pain	43 (36.4)	4 (40.0)
Chronic fatigue	18 (15.3)	1 (10.0)
**Management**$^{\Delta}$		
Number of drugs tried, median [IQR, range]	2 [1 – 3, 0 – 7]	1 [1 – 3, 0 – 5]
No treatment or simple analgesia only, n (%)	7 (5.9)	1 (10.0)
Colchicine, n (%)	110 (93.2)	9 (90.0)
Corticosteroid, n (%)	68 (57.6)	4 (40.0)
Anakinra, n (%)	16 (13.6)	2 (20.0)
Disease modifying anti-rheumatic drug(s), n (%)	23 (19.5)	2 (20.0)
Other biologic drug, n (%)	3 (2.5)	0 (0.0)
Pericardiectomy	1 (0.7)	0 (0.0)
**Treatment of presenting episode, n (%)**$^{\Delta}$		
No treatment or simple analgesia only	71 (60.2)	7 (70.0)
Colchicine only	23 (19.5)	1 (10.0)
Corticosteroid only	20 (17.0)	1 (10.0)
Colchicine and corticosteroid	4 (3.4)	1 (10.0)

$^{\chi}$*Variants classified as pathogenic/ likely pathogenic or predicted to be pathogenic in silico (minor allele frequency <0.01, CADD >10).*

**Ancestry categorisation was performed to align with the groups defined on the Genome Aggregation Database (gnomAD)*

$^{\Psi}$*First degree relative with one or more episode(s) of confirmed acute pericarditis.*

[±] *Myocardial involvement is defined as a raised Troponin I/ T, or myocardial inflammation on cardiac MRI. Corticosteroid dependence is defined as requiring continuous daily corticosteroids for six months or longer. Chronic chest pain is defined as chest pain in the presence of normal investigations (inflammatory markers +/- ECG and imaging) on 2 or more occasions.*

[Δ] *Disease modifying drugs comprise colchicine, corticosteroid, anakinra, disease modifying anti-rheumatic drug or other biologic drug. Simple analgesia includes non-steroidal anti-inflammatory drugs.*

Table S7. Burden association test of rare synonymous variants in individuals of European (NFE) descent.

Gene	*Combined frequency of rare synonymous variants*		*p value*
	Allele frequency in cases	***Allele frequency in controls****	
MEFV	2.0% (4/200)	1.6% (2031/129198)	0.386

*This table shows the combined allele frequency of rare synonymous variants (number of variants divided by the total number of alleles) among the 100 NFE IRP cases and the 64,600 ancestry matched controls taken from the gnomAD database; comparison in made using a one-sided Fisher's test and values <0.05 are considered statistically significant. *Unselected controls comprise exomes/genomes of NFE individuals listed on the genome aggregation database (gnomAD v2.1.1). MAF: minor allele frequency; NFE: non-Finnish European.*

Figure S1. Recruitment and follow up of individuals with IRP by year.

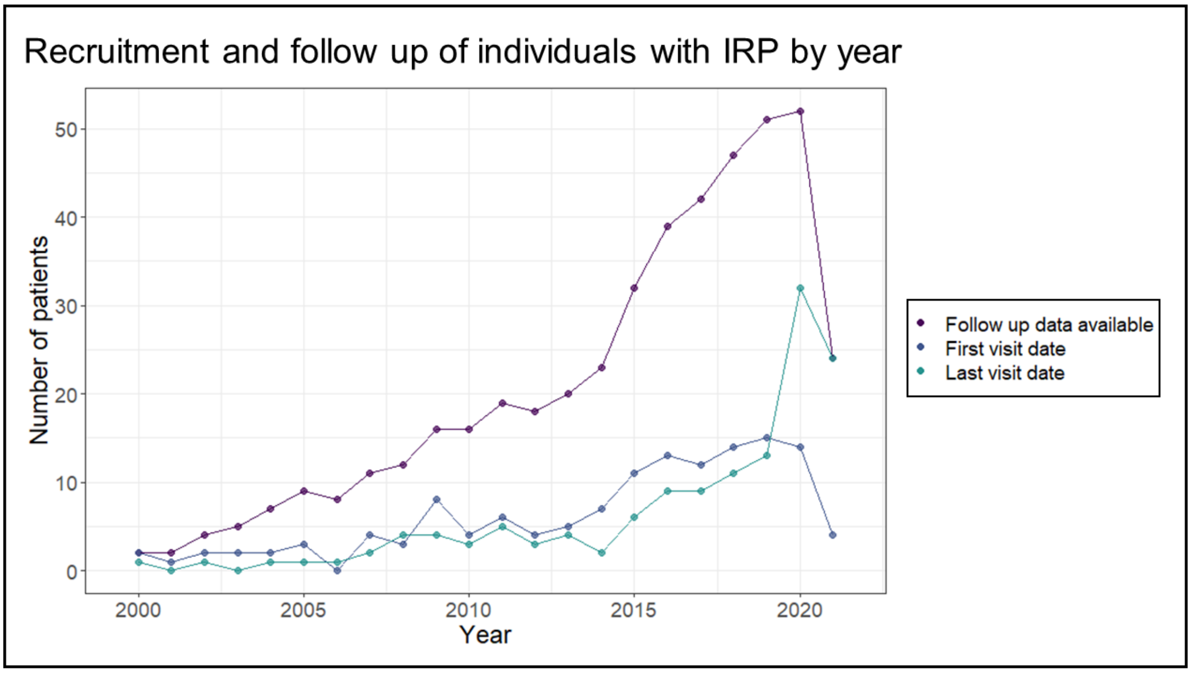

Recruitment and follow up of individuals with IRP by year. Line graph showing the number of cases (A) where follow up data was available in each year (B) that had their first visit in each year and (C) that had their most recent (last) study visit in each year.
